# Evaluation of the effects of cementless total hip replacement on femoral length in skeletally immature dogs

**DOI:** 10.1111/vsu.14180

**Published:** 2024-10-28

**Authors:** Ida Forzisi, Aldo Vezzoni, Luca Vezzoni, Dario Drudi, Alexandros Bourbos, Denis J. Marcellin‐Little

**Affiliations:** ^1^ Clinica Veterinaria Vezzoni Cremona Italy; ^2^ Department of Surgical and Radiological Sciences and the JD Wheat Veterinary Orthopedic Research Laboratory, School of Veterinary Medicine University of California, Davis Davis California USA

## Abstract

**Objective:**

To describe percentage length changes in the femur after total hip replacement (THR) performed before skeletal maturity.

**Study design:**

Retrospective study.

**Animals:**

Twenty‐four dogs younger than 8.5 months which underwent unilateral THR and had radiographic follow up.

**Methods:**

Preoperative and follow‐up radiographs were reviewed. Radiographic measurements included the length of the greater trochanter, femoral diaphysis and distal epiphysis, width of the femur 10 mm distal to the distal aspect of the greater trochanter, width of the femur at 50%, and femoral condylar offset. Percentage changes in length over time were compared among operated and contralateral femurs used as controls. Measurements were collected in triplicate in 10 dogs to evaluate consistency.

**Results:**

All repeated measurements had excellent consistency. The percentage increase in length of the greater trochanter was smaller in operated femurs than controls (mean difference: −11.5%, *p* = .017), but no differences were observed for the femoral diaphysis and distal epiphysis (−1.0%, *p* = .595), or the femur overall (−2.3%, *p* = .232). The percentage increase in femoral cortical width was greater in operated femurs than controls, both 10 mm distal to the greater trochanter (4.6% difference, *p* = .037) and at 50% length (8.5% difference, *p* = .030).

**Conclusion:**

In growing dogs, cementless THR decreased trochanteric growth by approximately 10% but did not change diaphyseal growth and femoral growth.

**Clinical significance:**

Cementless THR performed in skeletally immature dogs with severe hip problems did not impact femoral length in a clinically relevant fashion.

## 1 INTRODUCTION

Canine hip dysplasia is a widespread and most often bilateral orthopedic disease, affecting predominantly medium to large‐breed dogs.^1^ Total hip replacement (THR) is a well established surgical procedure considered to be the gold standard for management of debilitating conditions of the coxofemoral joints in dogs.^2^, ^3^, ^4^, ^5^ Osteoarthritis secondary to hip dysplasia is the most common indication for THR but other indications include the management of capital physeal fractures, developmental and traumatic hip luxation, and avascular necrosis of the femoral head, which occur in growing dogs.^6^, ^7^, ^8^, ^9^ The success rate of THR in growing dogs has been high and is comparable to the success rate of THR in adult dogs.^10^, ^11^ However, information in the scientific literature regarding the influence of THR on femoral growth and femoral geometry is limited.

An evaluation of changes in femoral length in skeletally immature dogs undergoing THR is warranted. This study evaluated changes in percentage length over time in skeletally immature femurs that underwent unilateral THR. Contralateral femurs were used as controls. We hypothesized that THR decreased femoral longitudinal and circumferential femoral percentage changes in length. We also hypothesized that the deficit in length caused by THR was greater in dogs operated at a younger age.

## 2 MATERIALS AND METHODS

### 2.1 Sample

This was a retrospective study with a convenience sample. Dogs that were 4 months old or older and 8.5 months old or younger, which underwent unilateral Kyon Zurich hip THR (Movora, Zürich, Switzerland) between April 2011 and February 2022 at the Clinica Veterinaria Vezzoni (Cremona, Italy), were eligible for inclusion. Dogs were excluded if radiographs of the operated and contralateral femurs before surgery and more than 3 weeks after surgery were unavailable.

### 2.2 Data collection

**FIGURE 1:**
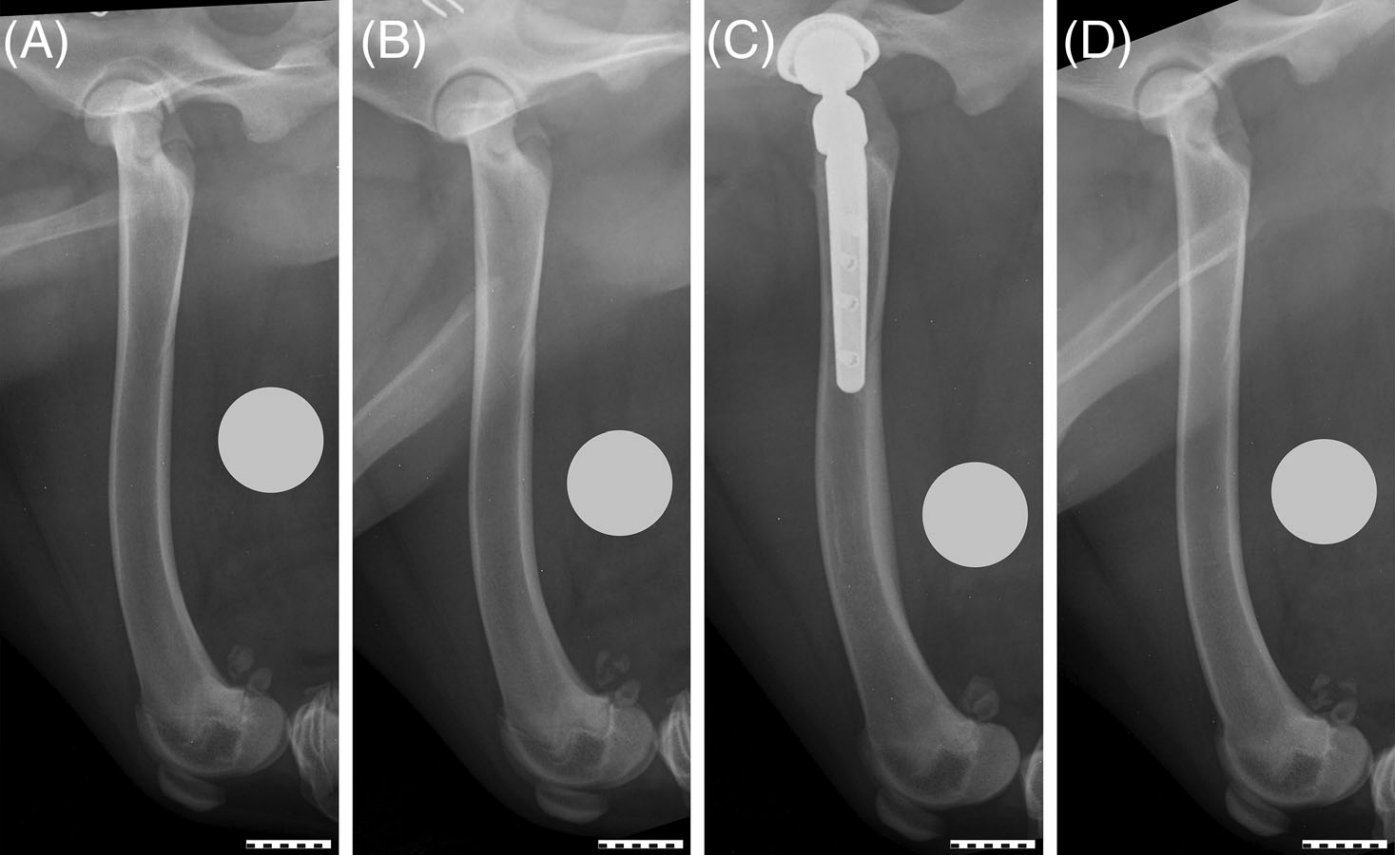
In mediolateral radiographic projections of the left (A) and right femur (B) of a 6.6‐month‐old Labrador retriever taken on the day of the left total hip replacement (THR), femoral lengths were 166.4 mm on the left and 169.0 mm on the right. At the 8.7 month reevaluation (63 days post‐THR), the operated right femur (C) measured 173.4 mm and the control femur (D) measured 174.7 mm. The 25 mm ball magnification markers were repositioned during figure preparation to facilitate cropping. Line bars = 20 mm.

**FIGURE 2:**
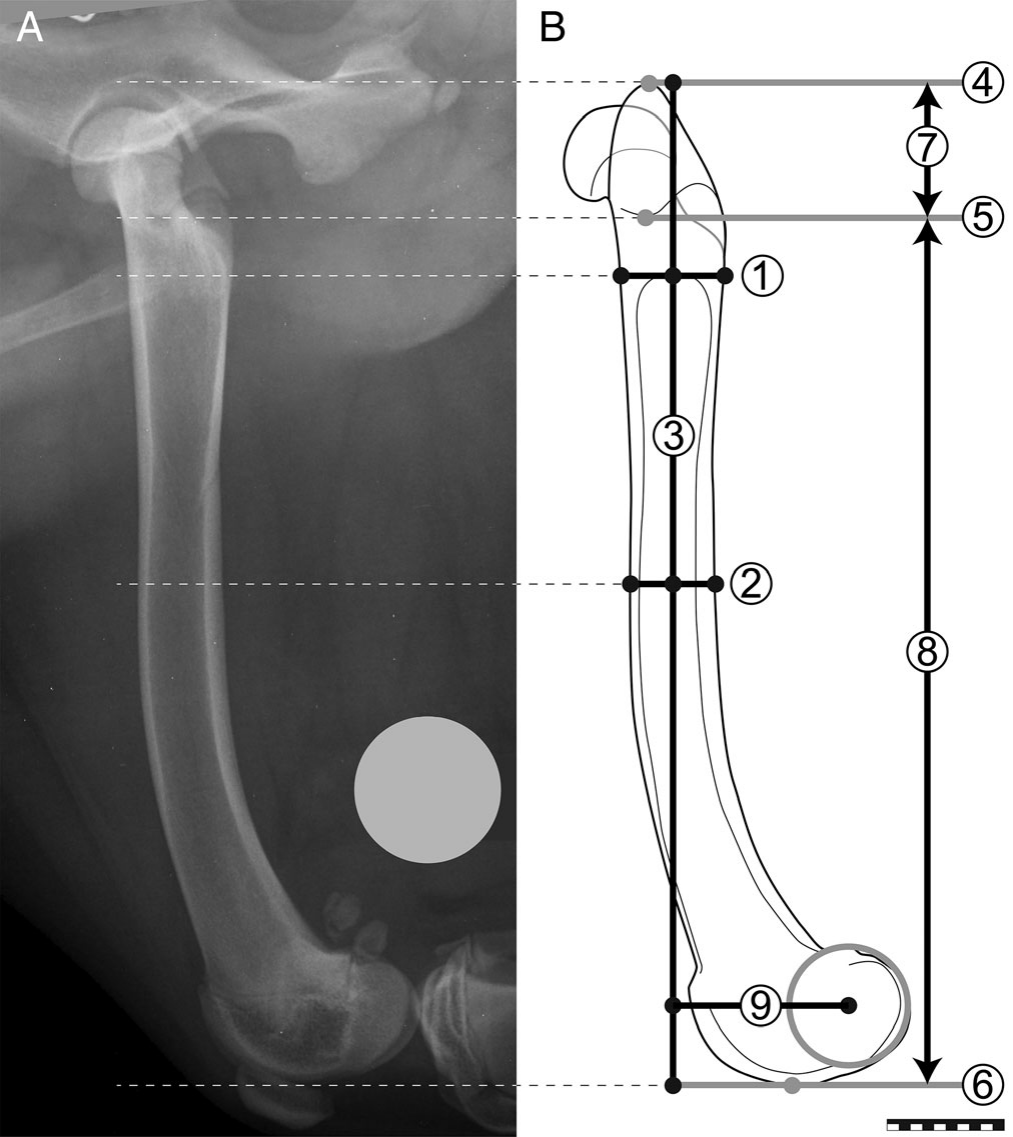
Mediolateral radiographic projection of the left femur shown in Figure 1 (A) and corresponding illustration (B) showing measurements of femoral length and width. The craniocaudal femoral width 10 mm distal to the distal aspect of the trochanter (line 1) and at 50% of femoral length (line 2) are shown. The centerline of the femoral shaft (line 3) is the line crossing the midpoint of lines 1 and 2. Lines perpendicular to line 3 are tangential to the proximal aspect of the greater trochanter (line 4), at the base of the trochanteric fossa (line 5), and tangential to the distal aspect of the femoral condyles (line 6). The length of the trochanter (line 7) is the distance between lines 4 and 5; the length of the femoral diaphysis and distal epiphysis (line 8) is the distance between lines 5 and 6. Femoral length (line 3) is the sum of the length of lines 7 and 8. A circle is fitted to the femoral condyles. Femoral condylar offset length (line 9) is the length of the line perpendicular to line 3, which starts on line 3 and ends at the center of the femoral condylar circle. The 25 mm ball magnification marker was repositioned during figure preparation to facilitate cropping. Line bar = 20 mm.

Breed, sex, age at surgery, and body weight were collected. Mediolateral radiographic projections were collected of the operated and contralateral femur before surgery and at the longest follow up (Figure 1). Radiographic measurements were collected by one investigator (IF) using digital software (vPoP PRO, Shrewsbury, United Kingdom). Measurements were collected in triplicate for 10 dogs to evaluate measurement consistency. One week lapsed between each set of repeated measurements. Radiographs were calibrated for size using a 25 mm ball magnification marker.^12^ Greater trochanter length, femoral diaphyseal and distal epiphyseal length, femoral width 10 mm distal to the distal aspect of the greater trochanter and at 50% of femoral length, femoral condylar offset,^13^ and stem length were measured (Figure 2).

### 2.3 Statistical analysis

Analyses used SAS version 9.4 (SAS Institute, Cary, North Carolina). Descriptive statistics included age at surgery, preoperative dog body weight, sex and neuter status, preoperative trochanteric length, femoral diaphyseal and distal epiphyseal length, femoral length (i.e., the sum of trochanteric and diaphyseal and epiphyseal length), femoral width 10 mm distal to the distal aspect of the greater trochanter and at 50% length, femoral condylar offset, and postoperative percentage change in trochanteric length, femoral diaphyseal and distal epiphyseal length, femoral length, femoral width 10 mm distal to the distal aspect of the greater trochanter and at 50% length, and femoral condylar offset. To control for potential differences in positioning between reevaluation and postoperative radiographs on operated limbs, stem length was measured for both sets of radiographs. Measurements from reevaluation radiographs were normalized so that stem length was identical for both sets of radiographs. A correction of 1 / (1 + correction factor) was applied to all reevaluation measurements for all length measurements on reevaluation radiographs, where the correction factor was the ratio of (stem length at reevaluation − postoperative stem length) / postoperative stem length, so that stem length was identical on both sets of radiographs. To calculate the difference in normalized growth in operated and control femurs regardless of femur length, differences in percentage length change between operated and control femurs were calculated.

Normality was checked using the Shapiro–Wilk test. Data were considered normally distributed when *W* > 0.90 and *p* > .05. Data that were normally distributed are reported as means ± SDs. Data that were not normally distributed are reported as median (range). Intraobserver consistency was determined by calculating the intraclass correlation coefficient (ICC) for triplicate measurements on 10 limbs.^14^ Intraclass correlation coefficient values below .5 represented poor consistency, values greater than or equal to .5 and less than or equal to .75 represented moderate consistency, values greater than or equal to .75 and below .9 represented good consistency, and values of .90 or above represented excellent consistency.^15^ Intraobserver repeatability within a limb was calculated as the SD (specifically, the square root of the mean square error from the linear mixed model with dog as a random effect).

**FIGURE 3:**
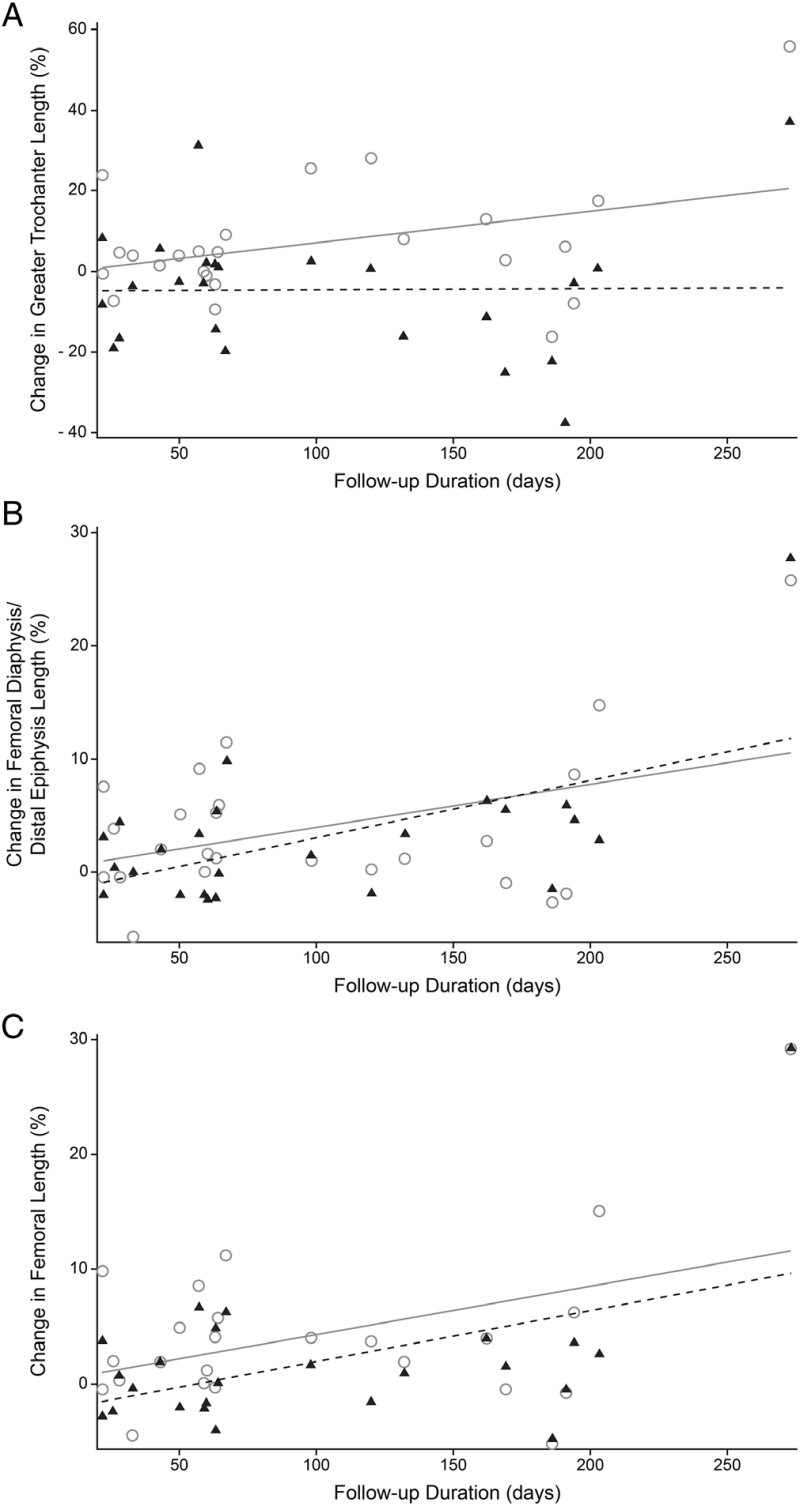
Linear regression plots showing percentage changes in length over time for the greater trochanter (A), the femoral diaphysis and distal epiphysis (B), and the femur (C) for 24 femurs that underwent a total hip replacement (black triangles) and contralateral femurs used as controls (open circles). The regression lines are shown for operated femurs (dashed lines) and controls (solid lines). Length increased over time in the femoral diaphysis and distal epiphysis (*R* ^2^ = .336, *p* = .003) and the femur (*R* ^2^ = .236, *p* = .016) but not in the greater trochanter (*R* ^2^ = .000, *p* = .965). Within each graph, the slope of the regression lines for the operated and control femurs do not differ statistically (*P* ranging from .234 to .916).

Measurement of percentage change in femoral length or width and body weight were compared using an ANOVA with preoperative/follow up and operated/contralateral (control) as categories, with repeated measured on dogs, body weight, and age as fixed effects, and percentage change in femoral length as a random effect. Similarly, changes between preoperative and follow‐up measurements of percentage change in body weight and in femoral length and width were analyzed using an ANOVA with preoperative/follow up and operated/contralateral (control) as categories, with repeated measured on dogs, body weight and age as fixed effects, and percentage change in femoral length as random effect. Effect sizes were calculated nonparametrically as abs(*Z*)/√*n*, where Z was the Wilcoxon paired sample test statistic and *n* was the group size. Effect sizes were defined as negligible when below .2, small when .2 or more and .5 or less, moderate when over .5 and .8 or less, and large when greater than .8.^14^ Regression analysis was used to evaluate the association between follow‐up duration and percentage change in femoral length or width in operated and control femurs (Figure 3). Regression analysis was also used to evaluate the association of differences in percentage change in femoral length in the operated and control femur and age at surgery. For regression analysis, the age used to calculate the follow‐up duration was capped at 14 months, based on the assumption that no femoral growth would occur beyond 14 months of age. The regression slopes in operated and control limbs were compared statistically. Regression analysis was also used to evaluate the association of age at the time of surgery and percentage change in femoral length and width. Statistical significance was defined as *p* < .05.

## 3 RESULTS

Eighty‐three dogs younger than 8.5 months underwent unilateral total hip replacement during the study period. Fifty‐nine dogs were excluded from the study because a THR in the contralateral pelvic limb was performed 3 weeks or less after the first THR or because radiographs were unavailable or were malpositioned. Twenty‐four dogs were included in the study. They underwent THR to manage luxoid hip dysplasia^16^ (*n* = 21) or a capital physeal fracture (*n* = 3). The dog breeds included the following: mixed‐breed dog (*n* = 7), German shepherd dog (4), Border collie (3), golden retriever (3), Labrador retriever (3) and one Australian shepherd dog, Cocker spaniel, lagotto Romagnolo, and a pit bull terrier. Ten dogs were male and 14 were female. The median age at surgery was 6.9 months with a range of 4.0 to 8.3 months. The median body weight was 20.8 kg with a range of 9.4 to 36.4 kg at surgery, and 25.0 kg with a range of 10.0 to 40.3 kg at follow up (Table 1). The median age at follow up was 9.5 months (with a range of 6.9–130.1 months) and the median follow‐up duration was 2.1 months (with a range of 0.7–123.4 months).

**TABLE 1:** Median (range) femoral geometry in 24 operated and contralateral femurs of skeletally immature dogs undergoing THR before surgery and at follow up.

	Preoperative assessment		Follow‐up assessment	
	Operated femur	Control femur	Operated femur	Control femur
Length (mm)				
Greater trochanter	24.4^1^ (17.2–31.7)	23.8^2^ (18.7–29.9)	22.0^1^ (14.9–39.7)	24.0^2^ (20.1–31.8)
Femoral diaphysis + epiphysis	158.3^a^ (102.2–188.7)	155.6^a^ (103.0–192.5)	159.4^b^ (100.7–195.0)	164.1^b^ (100.2–194.8)
Femur	181.9^a^ (121.4–214.9)	180.0^a^ (127.0–216.0)	182.9^b^ (115.6–219.3)	190.5^b^ (120.3–221.6)
Width (mm)				
10 mm distal to GT	16.2 (10.6–24.0)	16.2 (11.1–24.1)	16.8 (11.8–24.6)	16.5 (12.0–22.8)
50% length	15.0 (10.5–19.4)	15.1 (10.7–24.7)	16.2 (10.9–23.7)	15.3 (10.2–24.1)
Condylar offset	32.2^a^ (22.0–50.6)	33.3^a^ (18.1–49.4)	32.5^b^ (21.0–46.8)	33.5^b^ (22.4–51.6)
Body weight (kg)	20.8 (9.4–36.4)	20.8 (9.4–36.4)	25.0 (10.0–40.3)	25.0 (10.0–40.3)

*Note*: Group data were non‐normally distributed and are listed as medians (ranges). Within a row, median values with different superscript numbers differ (*p* < .05) between operated and control femurs at a given time point and median values with different superscript letters differ (*p* < .05) between preoperative and follow up both for operated and control femurs and for all femurs combined.

Abbreviations: GT, greater trochanter; THR, total hip replacement.

Preoperative measurements of trochanteric length (ICC = .975), femoral diaphyseal and distal epiphyseal length (ICC = .997), cortical width 10 mm distal to the greater trochanter (ICC = .998), cortical width at 50% length (ICC = .999), and femoral condylar offset (ICC = .998) showed excellent intra‐observer consistency. Preoperative measurement repeatability was 0.45 mm for greater trochanter length, 0.91 mm for diaphyseal and distal epiphyseal length, 0.13 mm for cortical width 10 mm distal to the greater trochanter, 0.08 mm for cortical width at 50% length, and 0.26 mm for femoral condylar offset. Postoperative measurements of trochanteric length (ICC = .995), femoral diaphyseal and distal epiphyseal length (ICC = .997), cortical width 10 mm distal to the greater trochanter (ICC = .996), cortical width at 50% length (ICC = .999), and femoral condylar offset (ICC = .999) also demonstrated excellent consistency. Follow‐up measurement repeatability was 0.27 mm for greater trochanter length, 0.97 mm for diaphyseal and distal epiphyseal length, 0.19 mm for cortical width 10 mm distal to the greater trochanter, 0.09 mm for cortical width at 50% length, and 0.26 mm for femoral condylar offset.

Before surgery, the greater trochanter was slightly longer in operated femurs (median, 24.4 mm) than controls (median, 23.8 mm, *p* = .039) but at the time of reevaluation, the greater trochanter was shorter in operated femurs (median, 22.0 mm) than controls (median, 24.0 mm, *p* = .002). Femoral diaphyseal and epiphyseal length, femoral length, and femoral condylar offset were larger at follow up than preoperatively in operated femurs and in controls (*p* ranging from .002 to .037). The percentage increase in length of the greater trochanter was smaller in operated femurs than controls (*p* = .002, Table 2). The percentage increase in length of the femoral shaft (*p* = .712), femur (*p* = .465), femoral cortical width 10 mm distal to the distal aspect of the greater trochanter (*p* = .203) and at 50% length (*p* = .076), and condylar offset (*p* = .647) did not differ among controls and operated femurs. The mean percentage change in length of operated femurs relative to control femur was 11.5% smaller for the greater trochanter, 1.0% smaller for the femoral diaphysis and epiphysis, 2.3% smaller for the femur overall, 4.6% greater for femoral cortical width 10 mm distal to the distal aspect of the greater trochanter, 8.5% greater for the femoral cortical width at 50% length, and 2.8% smaller for condylar offset (Table 2). Based on effect size calculations, THR had a moderate negative effect on percentage change in trochanteric length and a small negative effect on percentage change in femoral length. Conversely, THR had a moderate positive effect of increasing width 10 mm distal to the greater trochanter, a small positive effect of increasing width at 50% length. Total hip replacement had a negligible effect on percentage change in diaphyseal and distal epiphyseal length and condylar offset.

**TABLE 2:** Median (range) percentage change in femoral geometry over time in 24 operated and control femurs of skeletally immature dogs undergoing THR.

	Operated femur	Control femur	Difference^a^	Effect size of THR bone‐length changes
Length (% change)				
Greater trochanter	−2.5^A^ (−2.3 to 28)	1.8^B^ (−5.7 to 26)	−11.5% ± 15.1%	0.57 (moderate)
Femoral diaphysis + distal epiphysis	0.8 (−4.7 to 29)	2.9 (−5.2 to 29)	−1.0% ± 4.7%	0.12 (negligible)
Femur	−2.8 (−37 to 37)	4.3 (−16 to 27)	−2.3% ± 3.5%	0.35 (small)
Width (% change)				
10 mm distal to GT	−0.3 (−17 to 20)	−0.4 (−16 to 27)	4.6% ± 7.0%	0.58 (moderate)
50% length	4.2 (−2.7 to 64)	2.0 (−4.6 to 35)	8.5% ± 13.0%	0.44 (small)
Condylar offset	5.6 (−8.2 to 24)	0.8 (−8.7 to 33)	−2.8% ± 11.0%	0.08 (negligible)

*Note*: Group data were non‐normally distributed and are listed as medians (ranges). Within a row, median values with different superscript letters differ (*p* < .05) between operated and control femurs.

Abbreviations: GT, greater trochanter; THR, total hip replacement.

^a^ M ± SD of differences between operated and control limbs. These differences were normally distributed.

For regression analysis, associations were present between follow‐up duration and percentage change in femoral diaphyseal and epiphyseal length (*R* ^2^ = .336, *p* = .003, Figure 3) and between follow‐up duration and percentage change in femoral length (*R* ^2^ = .236, *p* = .016). Percentage changes in trochanteric length, width 10 mm distal to the greater trochanter and at 50% length, and femoral condylar offset were not associated with follow‐up duration (*p* ranging from .422 to .965). The slopes of femoral growth parameters in operated and control femurs did not differ (*p* ranging from .234 to .916). Associations between age at surgery and the differences in percentage length changes for the operated femur relative to the control femur were not identified for any of the parameters (*p* ranging from .462 to .745).

## 4 DISCUSSION

This study evaluated the percentage changes in femoral length and width in 24 dogs undergoing unilateral THR before skeletal maturity. All dogs were younger than 8.5 months of age at the time of surgery. An enrollment cut off of 8.5 months was selected because, subjectively, most skeletally immature patients with severe hip dysplasia that presented for THR appear to have undergone THR by 8.5 months of age. A later cut off could have led to the inclusion of dogs with minimal residual growth, negatively impacting the authors' ability to detect the impact of THR on percentage changes in femoral length. Total hip replacement had the effect of decreasing trochanteric growth by approximately 10% but did not appear to decrease growth of the femoral diaphysis and distal epiphysis. We therefore accepted the hypothesis that THR decreased femoral longitudinal growth. That decrease in growth, however, only affected the greater trochanter and would likely have no clinical impact.

While direct trauma to the greater trochanter was not observed during THR surgery, interference with trochanteric growth was possibly associated with drilling and rasping the proximal portion of the femur at the base of the greater trochanter. Trochanteric growth might have been impacted negatively by damage to the physis, by vascular changes following THR, or by the bone response to regional inflammation. Radiographically, the greater trochanteric physis reportedly closes between 6 and 11 months of age, the capital physis between 6 and 12 months of age, and the distal femoral physis close between 6 and 11 months of age.^17^ However, an author suggested in a review that the age range for closure of the greater trochanter physis was 9–11 months, while the age ranges for closure of the capital physis and distal femoral physis were 6–9 months and 6–8 months, respectively, suggesting that more residual growth could be present in the greater trochanteric physis than the capital physis at a given time.^18^ Physeal closure as seen on radiographs, however, might differ from functional growth at a physis. Regardless, damage to the greater trochanteric physis during the THR could have negatively impacted the remaining growth of the greater trochanter. Alternatively, canine cementless total hip replacement has been shown to alter femoral blood flow, with a potential negative influence bone growth.^19^, ^20^, ^21^ Inflammatory mediators such as tumor necrosis factor *α*, interleukin 1*β*, and interleukin 6 promote growth plate apoptosis and negatively impact growth.^22^ These inflammatory mediators could be present in response to THR. The relatively low negative impact of surgery on longitudinal bone growth is probably linked to the fact that the distal femoral physis is reportedly responsible for three quarters of the femoral longitudinal growth in dogs and one would not expect a THR to have negative effect on the distal femoral physis.^23^ Total hip replacement surgery could also have some trophic effect on the femur, similar to the few millimeters of bone overgrowth observed in the femur and ipsilateral tibia after a femoral fracture in children.^24^

Cortical width 10 mm distal to the distal aspect of the greater trochanter and at 50% femoral length increased after THR. We rejected the hypothesis that THR decreases circumferential femoral growth. This finding was unexpected. The increase in femoral width was likely due to a periosteal reaction and cortical hypertrophy secondary to THR, because of altered stress distributions in the femoral shaft.^25^ In a long‐term evaluation of 37 cementless total hip replacement, cortical hypertrophy was observed in approximately one third of femoral diaphyses.^4^ It is also possible that femoral reaming induced a cortical hypertrophy.^26^

This study had limitations. Negative length changes were the likely consequence of a lack of measurement precision, when measurements errors were larger than length changes. These negative measurements were more common for shorter measurements—that is, trochanteric length. The precision of femoral length measurements was negatively impacted by the fact that these measurements were the sum of measurements of trochanteric length and femoral shaft length. The sample of convenience was relatively small due to the fact that most dogs undergoing THR before skeletal maturity have severe hip problems including juvenile developmental hip luxation and most dogs with severe developmental hip problems are bilaterally affected.^27^ For dogs that underwent a second THR before skeletal maturity, the effect of THR on adult femoral length was unknown. The conclusions of the study, therefore, are limited to the evaluation of continued femoral growth after THR but do not apply to the ultimate femoral morphology in adult dogs that underwent THR before reaching skeletal maturity. While the changes identified in this study are unlikely to reverse themselves, the effects of THR on femoral geometry at adulthood cannot be determined from the study. Additional research evaluating the geometry and composition of femurs after THR is warranted, particularly when THR was performed before skeletal maturity.

The contralateral limb may not have been an optimal control after total joint replacement,^28^ in part because a postoperative weight shift could influence bone metabolism and because hip dysplasia was present in the majority of contralateral limbs. Little is known about the influence of hip dysplasia on femoral growth but, in one study, hip dysplasia delayed the median age of radiographic appearance of the center of ossification of the femoral head from 13 to 16 or 17 days and delayed the median age of radiographic closure of the capital physis from 207 to 234 days.^34^

Length measurements from radiographs to evaluate growth possibly lacked precision because of radiographic magnification and distortion and potential differences in positioning in radiographs made at separate timepoints.^30^ Inconsistency in positioning of the limb was likely. That inconsistency has been shown to influence measurements of the position of a cementless stem in dogs.^31^ To minimize the effects of positioning inconsistency on measurements of operated femurs, stem length was measured on postoperative and on reevaluation radiographs and reevaluations measurements were adjusted so that stem length was identical on postoperative and reevaluation radiographs. Potential inconsistency was concerning for measurements of the greater trochanter, where approximately half of the measurements were smaller at reevaluation compared to postoperative radiographs. As it seems biologically impossible that the greater trochanter decreased in length over time, negative measurements were most likely due to measurement errors. Negative measurements were small overall—most often a few percentage points. A 4% decrease in length for a 25 mm long greater trochanter would represent only a 1 mm decrease in measured length. It is therefore conceivable that, if growth of the greater trochanter was minimal after THR, negative readings would be common. Several factors may also have decreased the accuracy of measurements, the proximal aspect of the greater trochanter had a low radio‐opacity and its view was potentially obstructed by the femoral head, the appearance of the distal aspect of the greater trochanter and the trochanteric physis at the trochanteric fossa may have changed as the femurs were maturing, and potential interference of the stem with readings. The regression plots, which took the negative readings into account, showed slight growth of the trochanter in control femurs. Readings of greater trochanter length should therefore be interpreted with caution because negative measurements were common. Negative measurements were also observed for a few measurements of other geometric parameters. There sources were likely similar to the negative measurements of trochanteric length.

Based on high ICCs and repeatability, radiographic measurements were highly consistent. However, that does not guarantee their accuracy.

Other methods such as computed tomography scanning, magnetic resonance imaging, or biplanar radiography would have increased precision of the evaluation of bone growth.^32^, ^33^, ^34^ However, the use of these methods was not possible in the clinical environment where data were acquired.

The impact of the screwed‐on osseointegrated cementless THR stems placed in the study may differ from the impact of a press‐fit osseointegrated cementless THR stem because endosteal contact is likely more limited with a screwed‐on stem than a press‐fit stem. A retrospective study of press‐fit THR in 20 growing dogs reported cortical bone loss but did not report a growth deficit in operated dogs.^11^

We concluded from the current study that screwed‐on osseointegrated cementless THR can be performed in growing dogs with minimal impact on femoral morphology.

## FUNDING INFORMATION

This study was self‐funded by Clinica Veterinaria Vezzoni and University of California, Davis.

## CONFLICT OF INTEREST

Vezzoni L and Marcellin‐Little DJ are remunerated members of Movora's Global Advisory Board and Vezzoni A is a nonremunerated honorary member of Movora's Global Advisory Board. Movora is the manufacturer and distributor of the total hip replacement implants used in the study. The other authors have no financial or personal relationships that could inappropriately influence or bias the study content.
